# Mapping membrane lipids in the developing and adult mouse retina under physiological and pathological conditions using mass spectrometry

**DOI:** 10.1016/j.jbc.2021.100303

**Published:** 2021-01-16

**Authors:** Fumie Hamano, Hiroshi Kuribayashi, Toshiro Iwagawa, Asano Tsuhako, Katsuyuki Nagata, Hiroshi Sagara, Takao Shimizu, Hideo Shindou, Sumiko Watanabe

**Affiliations:** 1 Department of Lipid Signaling, National Center for Global Health and Medicine, Shinjuku-ku, Tokyo, Japan; 2 Life Sciences Core Facility, Graduate School of Medicine, University of Tokyo, Bunkyo-ku, Tokyo, Japan; 3 Division of Molecular and Developmental Biology, Institute of Medical Science, University of Tokyo, Minato-ku, Tokyo, Japan; 4 Medical Proteomics Laboratory, Institute of Medical Science, University of Tokyo, Minato-ku, Tokyo, Japan; 5 Department of Lipid Science, Graduate School of Medicine, University of Tokyo, Bunkyo-ku, Tokyo, Japan

**Keywords:** DHA, docosahexaenoic acid, DPPC, dipalmitoylphosphatidylcholine, EPA, eicosapentaenoic acid, LC, long chain, ONL, outer nuclear layer, OS, outer segment, PC, phosphatidylcholine, PE, phosphatidylethanolamine, PUFA, polyunsaturated fatty acid, RPE, retinal pigment epithelium, SRM, selected reaction monitoring, VLC, very long chain

## Abstract

Membrane phospholipids play pivotal roles in various cellular processes, and their levels are tightly regulated. In the retina, phospholipids had been scrutinized because of their distinct composition and requirement in visual transduction. However, how lipid composition changes during retinal development remains unclear. Here, we used liquid chromatography–mass spectrometry (LC-MS) to assess the dynamic changes in the levels of two main glycerophospholipids, phosphatidylcholine (PC) and phosphatidylethanolamine (PE), in the developing mouse retina under physiological and pathological conditions. The total levels of PC and PE increased during retinal development, and individual lipid species exhibited distinct level changes. The amount of very-long-chain PC and PE increased dramatically in the late stages of retinal development. The mRNA levels of *Elovl2* and *Elovl4*, genes encoding enzymes essential for the synthesis of very-long-chain polyunsaturated fatty acids, increased in developing photoreceptors. Cell sorting based on CD73 expression followed by LC-MS revealed distinct changes in PC and PE levels in CD73-positive rod photoreceptors and CD73-negative retinal cells. Finally, using the NaIO_3_-induced photoreceptor degeneration model, we identified photoreceptor-specific changes in PC and PE levels from 1 day after NaIO_3_ administration, before the outer segment of photoreceptors displayed morphological impairment. In conclusion, our findings provide insight into the dynamic changes in PC and PE levels in the developing and adult mouse retina under physiological and pathological conditions. Furthermore, we provide evidence that cell sorting followed by LC-MS is a promising approach for investigating the relevance of lipid homeostasis in the function of different retinal cell types.

The vertebrate retina consists of six types of neurons and Müller glia. In mice, these major retinal cell types originate from common retinal progenitor cells and are developed between embryonic day (E) 11 and postnatal day (P) 10 in a conserved temporal order (1). The majority of retinal ganglion cells, amacrine cells, horizontal cells, and cone photoreceptors differentiate before birth, whereas bipolar cells, rod photoreceptors, and Müller glia are largely generated after birth. Numerous studies have shown that the well-ordered retinal cell differentiation is regulated by the coordinated action of transcription factors and epigenetic mechanisms (2, 3).

Retinal photoreceptor degeneration is a leading cause of blindness. Despite extensive efforts, there is currently no effective treatment for retinal photoreceptor degeneration. Various biological processes, such as lipid metabolism, have been suggested to regulate photoreceptor maintenance. Of note, mounting evidence suggests that dysregulation of lipid homeostasis leads to retinal degeneration. Rd1-16 mice have widely been used as mouse models of inherited retinal degeneration and to study the mechanisms of retinitis pigmentosa. Rd11 mice have a single-nucleotide insertion (c.420-421 in sG) in exon 3 of the *Lpcat1* gene (4). LPCAT1 is a phospholipid biosynthesis/remodeling enzyme that catalyzes the conversion of palmitoyl-lysophosphatidylcholine into dipalmitoylphosphatidylcholine (DPPC) (5). Although reduced DPPC levels were found in the retina of rd11 mice (4), the mechanistic links between low DPPC levels and photoreceptor degeneration remain unclear.

Omega-3 long-chain polyunsaturated fatty acids (LC-PUFAs) exert antiangiogenic and neuroprotective functions in the retina (6). The LC-PUFA docosahexaenoic acid (DHA) plays a pivotal role in photoreceptor development and maintenance (7, 8). High intake of eicosapentaenoic acid (EPA) and DHA has been shown to prevent or delay intermediate age-related macular degeneration. However, the role of EPA and DHA in advanced age-related macular degeneration remains unknown (9). We previously found that mice lacking lysophosphatidic acid acyltransferase 3 (LPAAT3), a DHA-containing phospholipid biosynthetic enzyme, exhibited retinal development impairments, such as incomplete elongation of the outer segment (OS) and degeneration characterized by a decreased thickness of the outer nuclear layers (ONLs), as well as impaired visual function and disordered disc morphology in photoreceptor cells (10). Stargardt's disease is characterized by the accumulation of lipofuscin in the retinal pigment epithelium (RPE), leading to photoreceptor degeneration (11). Mutations in the elongase-coding gene *Elovl4*, which is essential for very-long-chain PUFAs (VLC-PUFAs), have been implicated in the development of a rare form of Stargardt's disease known as Stargardt's disease 3 (12, 13). In addition, mutations in ABCA4, an ATP-binding cassette transporter facilitating the removal of potentially reactive retinal lipid derivatives, have been implicated in Stargardt's disease pathology (14, 15). These data support the importance of lipid homeostasis in preventing retinal degeneration.

Recent evidence suggested the involvement of aging and stress in neuronal degeneration in the brain; changes in lipid composition have been proposed as a critical link between aging and neuronal degeneration. Although age-related changes in retinal glycopeptidolipid are insignificant (16), stress-induced dysregulation of lipid homeostasis may promote photoreceptor degeneration.

Lipid composition mapping in the retina in health and disease is important for the elucidation of the relevance of lipid homeostasis in retinal degeneration. Previous work had identified dynamism of phospholipids during retinal development (17). In this study, we focused on changes in the levels of phosphatidylcholine (PC) and phosphatidylethanolamine (PE), the most abundant phospholipids in mammalian cell membranes (18). We examined the temporal changes in PC and PE composition in the developing retina using liquid chromatography–mass spectrometry (LC-MS). In combination with our technique of retinal cell fractionation, we performed a comparative analysis of PC and PE levels in photoreceptors and other retinal cells. Our data revealed dynamic changes in PC and PE composition depending on the developmental stage, retinal cell lineage, and pathological situation. Cell sorting may serve as a powerful tool to study membrane lipid biology in the retina.

## Results

### Changes in membrane phospholipids in the developing mouse retina

**Figure 1:**
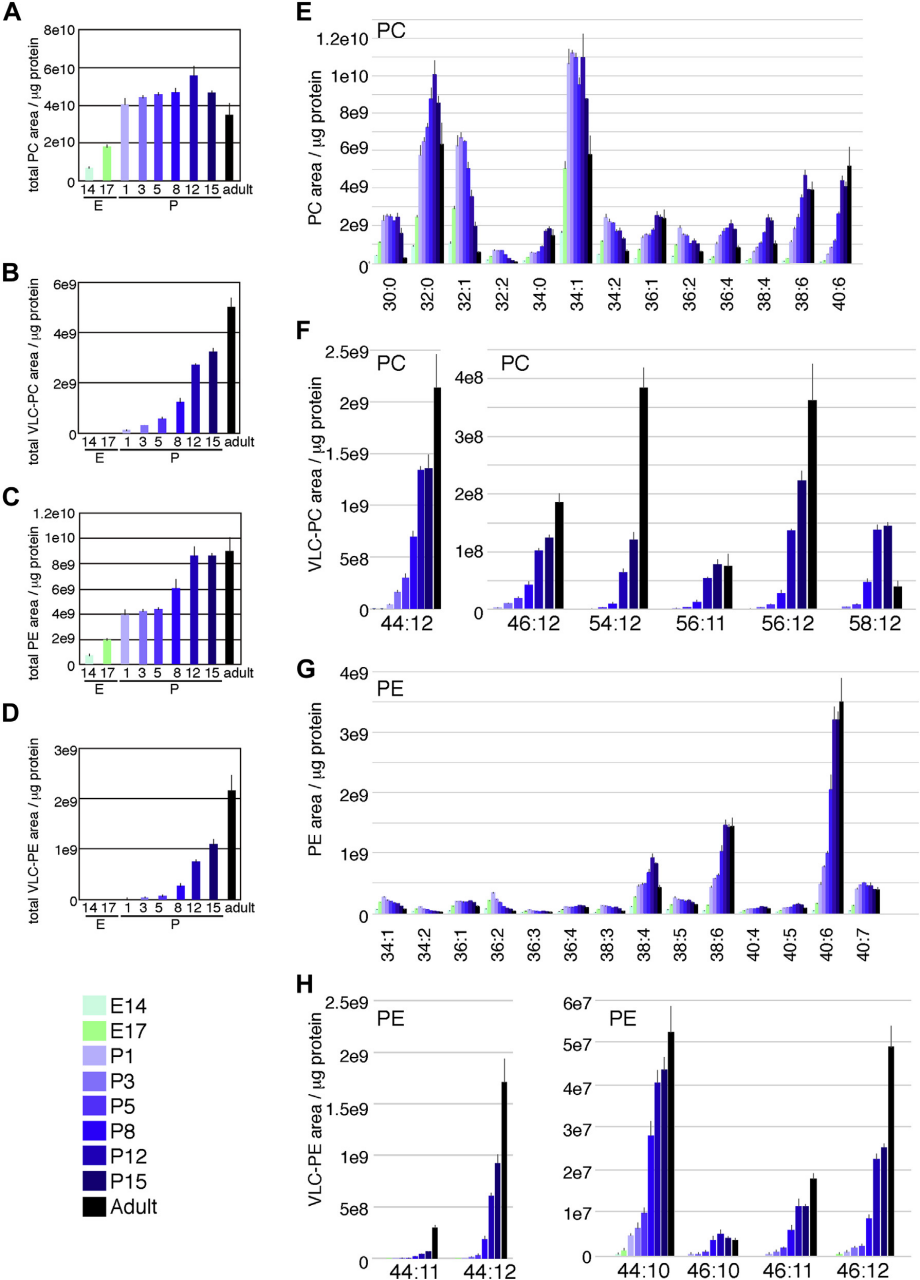
**Transition of PC and PE components during retinal development.** Mouse retinas at different developmental stages were isolated, and the PC and PE composition was analyzed by LC-MS. *A–D*, show total areas of PC (*A*), VLC-PC (*B*), PE (*C*), VLC-PE (*D*) per microgram protein. VLC-PC and VLC-PE are defined as PC and PE with molecular weight more than 44:12. Total areas of PC and PE are the sum of 238 species. *E–H*, area of each species per microgram protein is shown. Values are average of three to four independent samples with standard deviation. Two retinas were used to prepare one sample in all developmental stages except for the adult stage retina, which used one retina for one sample. PC, phosphatidylcholine; PE, phosphatidylethanolamine; VLC, very long chain.

To examine the changes in PC and PE levels during retinal development, we isolated the retinas of mice at different developmental stages ranging between E14 and 8 weeks old (adults). Membrane lipids were extracted and subjected to LC-MS to measure the levels of PC and PE. Protein amounts were measured, and the total PC or PE peak-area per microgram protein was calculated. The total PC area/protein in the retina sharply increased from E14 until birth, followed by a slow increase until P12 and then a decrease (Fig. 1*A*). Increasing evidence shows that VLC-PUFAs are critical regulators of neuronal survival (13). In the retina, VLC-PUFAs are enriched in light-sensitive photoreceptors in the outer retinal segments (7, 13, 19). Here, we defined VLC-PC and VLC-PE as PC and PE species having more than 44:12 molecular weight. Although VLC-PC levels were negligible in the embryonic period, they dramatically increased after birth (Fig. 1*B*). The total amount of PE/protein also increased until birth, and then stayed constant until P5. Subsequently, the total amount of PE/protein sharply increased until P12 and then stayed constant until adulthood (Fig. 1*C*). The changes in the total amount of VLC-PE (Fig. 1*D*) followed a similar pattern to that of total VLC-PC (Fig. 1*B*).

We then examined the levels of individual PC species during retinal development. PC 32:0 and 34:1 were abundant PC species; their levels increased in the embryonic period, followed by a decrease after birth (Fig. 1*E*). All other PC species exhibited dynamic and distinct temporal patterns during retinal development, and the levels of most of them increased during the embryonic period (Fig. 1*E*). The levels of VLC-PC exhibited a continuous and sharp increase after birth, especially after P12 (Figs. 1*F* and S1). This observation is consistent with the OS development in the late phase of retinal development. However, the levels of certain PC species, including 52:6, 56:10, 58:11, and 58:12, peaked at P15, and their levels dramatically decreased in the adult retina (Figs. 1*F* and S1)

The levels of predominant PE species in the retina, such as 38:4, 38:6, and 40:6, showed a continuous increase during retinal development until P12 (Fig. 1*G*), largely accounting for the observed pattern of total PE during retinal development (Fig. 1*C*). The levels of other PE species peaked around birth, followed by a gradual decrease (Fig. 1*G*). The levels of the primary VLC-PE molecular species, 44:12 and 44:11, increased after birth (Fig. 1*H*).

### Membrane phospholipid composition in the degenerating retina of rd1 mice

**Figure 2:**
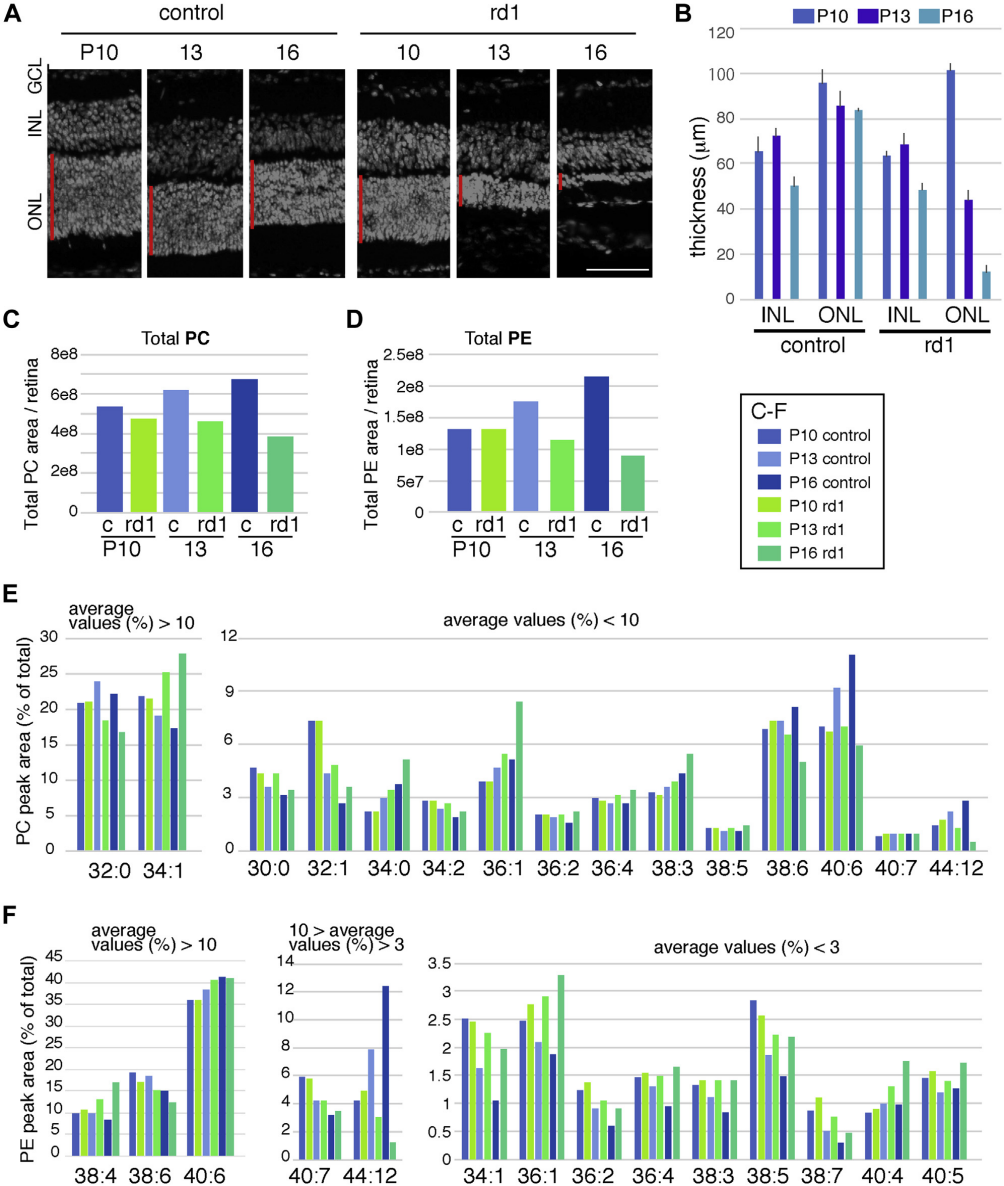
**Analysis of PC and PE components of rd1 retinal degeneration model mice.***A* and *B*, frozen sectioned retinas of control and rd1 mice retinas at P10, P13, and P16 were frozen sectioned, and nuclei were visualized with 4′,6-diamidino-2-phenylindole staining. The scale bar represents 50 μm. The thickness of the inner nuclear layer and outer nuclear layer was measured, and average of three samples with standard deviation is shown (*B*). *C–F*, the retinas of control or rd1 mice at P10, P13, and P16 were harvested, and contents of PC and PE were analyzed. *C* and *D*, total PC (*C*) and PE (*D*) areas of control of rd1 mice–derived retina are shown. Total areas of PC and PE are the sum of 266 and 273 species, respectively. Area of each species to the total area (%) of PC (*E*) and PE (*F*) are shown. The left panes show species with average values more than 10, and the right panel of E shows species with average values less than 10. The middle panel of F shows average more than 3 as well as less than 10, and the right panel shows average less than 3. PC, phosphatidylcholine; PE, phosphatidylethanolamine.

Next, we examined membrane phospholipid composition in the degenerating retina of rd1 mice. Previous works that examined the alteration of fatty acid composition and DHA metabolism in mouse retina of wildtype and rd1 by TLC or TLC/GC methods showed altered fatty acid metabolism especially an increment of DHA during retinal development (17, 20). In the rd1 mouse model, photoreceptor degeneration starts at around P10 (21, 22) and progresses rapidly; at P16, only a thin ONL was observed in the retina (Fig. 2, *A* and *B*). In contrast, the thickness of the inner nuclear layer was indistinguishable between control and rd1 retinas in these three developmental stages (Fig. 2*B*). We isolated the retinas of rd1 and control mice at P10, P13, and P16 and subsequently analyzed the PC and PE components. The levels of both total PC and PE per retina decreased in the developing retina of rd1 mice; in contrast, the levels of total PC and PE increased in the developing retina of control mice (Fig. 2, *C* and *D*). This is probably because the size of the rd1 retinas gradually decreased. In rd1 retinas, the levels of PC 32:0, PC 38:6, and PC/PE 44:12 decreased during development, although the levels of these lipid species increased in control retinas (Fig. 2, *E* and *F*). In contrast, the levels of PC 34:1 and PE 38:4 increased in the developing retina of rd1 mice but decreased in control retinas (Fig. 2, *E* and *F*).

### Analysis of PC and PE composition in fractionated retinal cells

The decrease in PC and PE levels in the developing rd1 retina could be explained by the progressive loss of photoreceptors; hence, we next measured the levels of photoreceptor-specific PC and PE after retinal cell sorting. First, we examined the effects of cell sorting on the membrane PC and PE composition. We also determined the number of cells required to measure PC and PE levels by LC-MS. We isolated the retinas of mice at E17 or adult mice, followed by trypsinization and cell sorting. Cells were collected, and membrane lipids were extracted after pooling different numbers of cells (Fig. S2*A*). The extracted lipids were subjected to LC-MS for PC and PE composition analysis. Total levels of PC or PE increased with increasing numbers of cells (Fig. S2, *B* and *D*). Values per 1 × 10^4^ cells showed larger but higher standard deviation values in samples with smaller number of cells than other samples in both PC and PE analyses (Fig. S2, *C* and *E*).

The predominant PC species exhibited comparable levels in E17 and adult retinas, with some exceptions (Fig. S2, *F* and *G*, left panel). It is surprising that this was also the case for low-abundance PC species (Fig. S2, *F* and *G*, right panel), indicating the high sensitivity of the method regardless of the cell number. In adult retinas, long-chain PC, such as PC 46:12 and PC 54:12, exhibited comparable levels from 5 × 10^3^ to 1 × 10^5^ cells, suggesting that a small number of cells was sufficient to analyze long-chain fatty acids (Fig. S2*G*, right panels). Similarly, the levels of most PE species in E17 and adult retinas showed comparable values, with a few exceptions when 5× 10^3^ cells were used for analysis (Fig. S2, *H* and *I*). However, many low-abundance PE species exhibited varying levels among different samples (data not shown).

As a control, lipid samples from E17 and adult retinas were also subjected to LC-MS at the same time with samples from sorted cells. In E17 retinas, we found similar PC and PE levels in sorted and whole retinal samples (Fig. S2, *F* and *H*). In adult retinal samples, we found comparable PC and PE levels for abundant species; however, low-abundance species exhibited higher relative values in sorted samples than in control samples (Fig. S2, *G* and *I*). Therefore, for subsequent experiments, we used 5 × 10^4^ cells.

### Analysis of PC and PE levels in CD73-positive rod photoreceptors and CD73-negative retinal cells

Rod photoreceptors comprise 70% to 80% of retinal cells; therefore, LC-MS analysis in whole retinal samples does not provide sufficient information on the lipid composition of less abundant retinal cells. We previously identified CD73 as a marker of rod photoreceptors in the developing and adult retina (23). RNA sequencing analysis of adult CD73-positive and CD73-negative retinal cells (GSE71462, GSE71464) (24) confirmed the expression of rod photoreceptor-specific genes in the CD73-positive fraction at P12 (Fig. S3).

**Figure 3:**
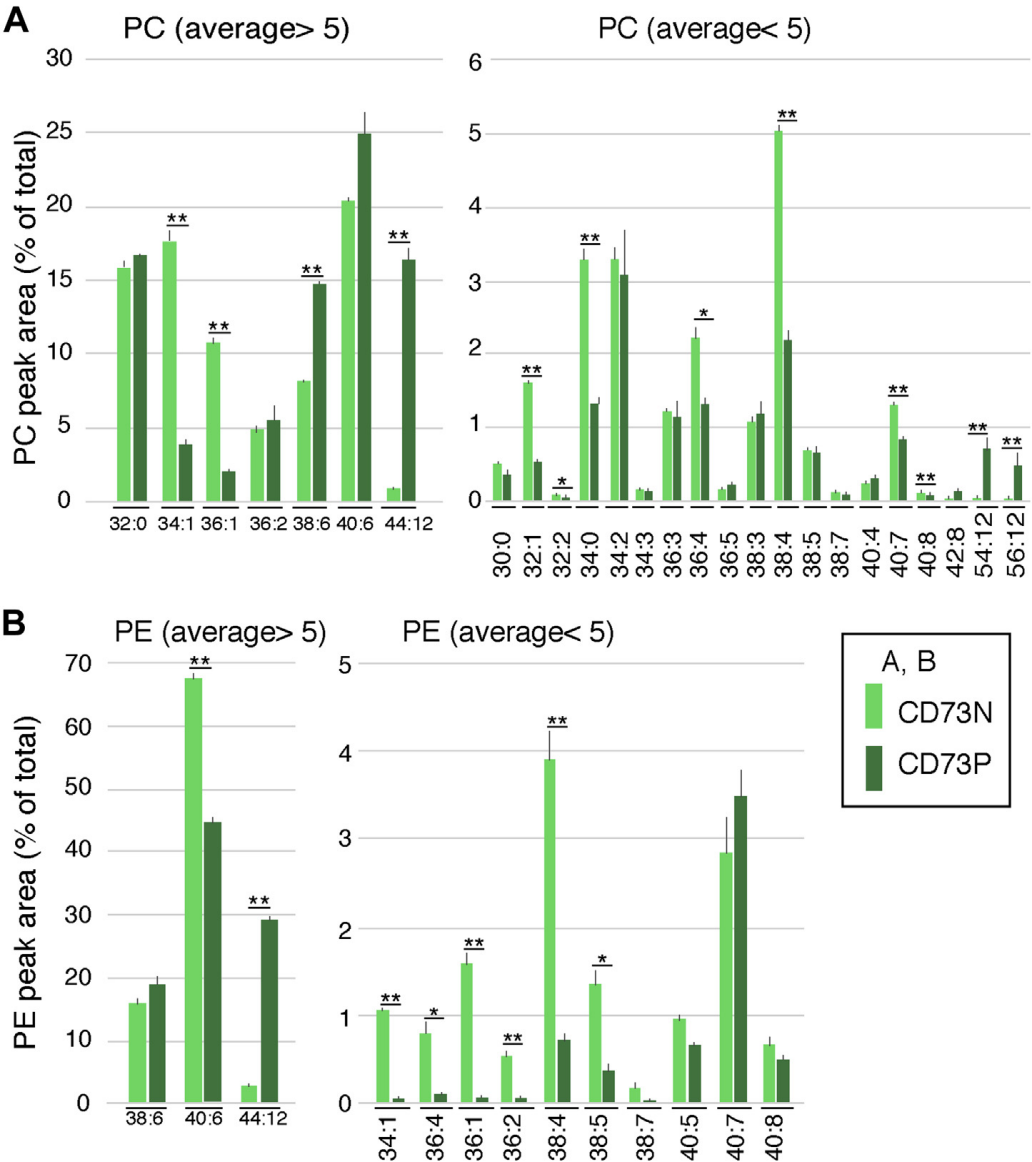
**Comparison of PC and PE components between CD73-positive rod photoreceptor and CD73-negative other retinal cells**. Adult retinal cells were fractionated into CD73-positive and -negative cells by the cell sorter. Then PC and PE components of both fractions were analyzed by LC-MS. For PC, 25 species were detected, and 14 species were detected for PE. The sum of 25 and 14 species of PC and PE, respectively, were used as total area values. Area of each species to the total area (%) of PC (*A*) and PE (*B*) is shown. The left panels are for species with average (%) values more than 5, and right panels show those under 5. The values are average of three independent samples with standard deviation. ∗*p* < 0.05, ∗∗*p* < 0.01, by unpaired *t* tests.

We analyzed PC and PE levels by LC-MS after sorting retinal cells based on CD73 expression. We observed striking differences in PC levels between CD73-positive and CD73-negative cells. Of note, PC 34:1 and PC 36:1 levels were considerably higher in CD73-negative than in CD73-positive cells, whereas the opposite was observed for PC 44:12, PC 38:6, and PC 40:6 (Fig. 3*A*), although the difference for PC 40:6 did not reach statistical significance. Among the low-abundance species, VLC-PC species (54:12, 56:12) were almost exclusively present in the CD73-positive cell population (Fig. 3*A*, right panel). Although PE 44:12 levels were significantly higher in CD73-positive cells, CD73-negative cells exhibited higher levels of most PE species (Fig. 3*B*).

### Dramatic changing of expression levels of lipid biosynthesis–related enzymes

**Figure 4:**
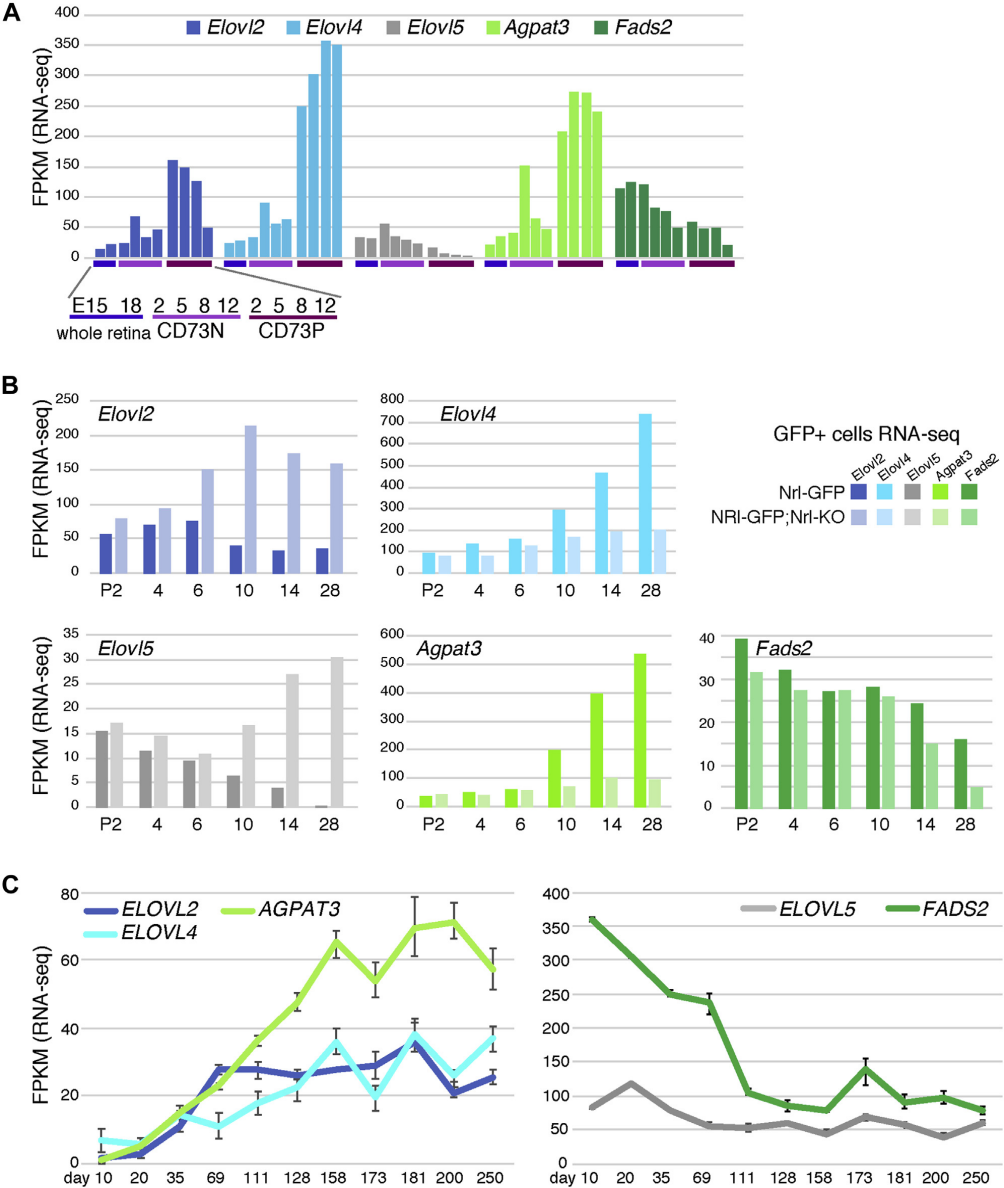
**The expression dynamics of lipid biosynthesis–related enzymes.***A*, RNA-Seq data of E15, E18 whole retina and CD73-positive rod photoreceptors and CD73-negative retinal cells at P2, P5, P8, and P12 (GSE71462, GSE71464). *B*, the expression profiling of flow-sorted rods from Nrlp-GFP mouse retina and GFP-positive S-cone-like cells from NRlp-GFP;Nrl-KO mouse (28, 29) (GSE74660, GSE74657). *C*, publicized RNA-Seq data of human iPS-derived retinal organoid (GSE119320) (31).

We then examined the dynamism of expression patterns of genes involving VLC during retinal development. ELOVL2 and ELOVL4 are elongases involved in VLC-PUFA-CoA biosynthesis (7, 25). *Elovl4* mRNA levels have been shown to increase during retinal development (26), especially in the photoreceptor inner segments (12). Using our previous RNA sequencing data (GSE71462, GSE71464) (24), we found that *Elovl2* and *Elovl4* were upregulated in CD73-positive rod photoreceptors as early as at P2, which corresponds to the rod photorecetpor birth period (Fig. 4*A*). During late retinal development, which corresponds to the rod photoreceptor maturation period, although *Elovl4* expression levels kept high in CD73-positive cells, *Elovl2* expression in CD73-positive cells gradually decreased and became similar to the level of the CD73-negative fraction (Fig. 4*A*). Although ELOVL5 was also shown to catalyze the elongation of long-chain fatty acids, *Elovl5* mRNA levels were higher in nonphotoreceptor cells than in rod photoreceptors (Fig. 4*A*). Consistently, no mutations in *Elovl5* were observed in patients with autosomal recessive retinitis pigmentosa (27). Nrl is the Maf-family leucine zipper transcription factor, and in the Nrl knockout (Nrl-KO) retina, photoreceptor precursors fated to cells with characteristics of short-wavelength cones (28). The expression profiling database of flow-sorted rods from Nrlp-GFP mouse retina (29) and GFP-positive S-cone-like cells from NRlp-GFP;Nrl-KO mouse (30) can be used to compare gene expression patterns between rod and S-cone (GSE74660, GFE74657). We confirmed that rod photoreceptor kept high *Elovl4* expression pattern after P14, which was in contrast to the low expression level of *Elovl2* (Fig. 4*B*). Of interest, Nrl-KO mice–derived retina showed higher expression levels of *Elovl2* and *Elovl5*, but Elovl4 was lower than Nrl-GFP rod (Fig. 4*B*), suggesting differential expression patterns of these genes between rod and cone.

We also examined two enzymes involved in DHA metabolism and found that *Agpat3* was expressed in CD73P cells much higher than in CD73N cells, but *Fads2* in CD73P cells was rather lower than in CD73N cells (Fig. 4*A*). The expression pattern of *Fads1* was similar to that of *Fads2* (data not shown). The Nrl-KO retina showed that the expression levels of both *Agpat3* and *Fads2* were lower in cone than in rod (Fig. 4*B*). Publicized RNA-Seq data of human iPS-derived retinal organoid (GSE119320) (31) showed similar expression transition of ELOVL members and *AGPAT3* and *FADS2* during development (Fig. 4*C*).

### PC and PE analyses of NaIO_3_-treated degenerating retina

**Figure 5:**
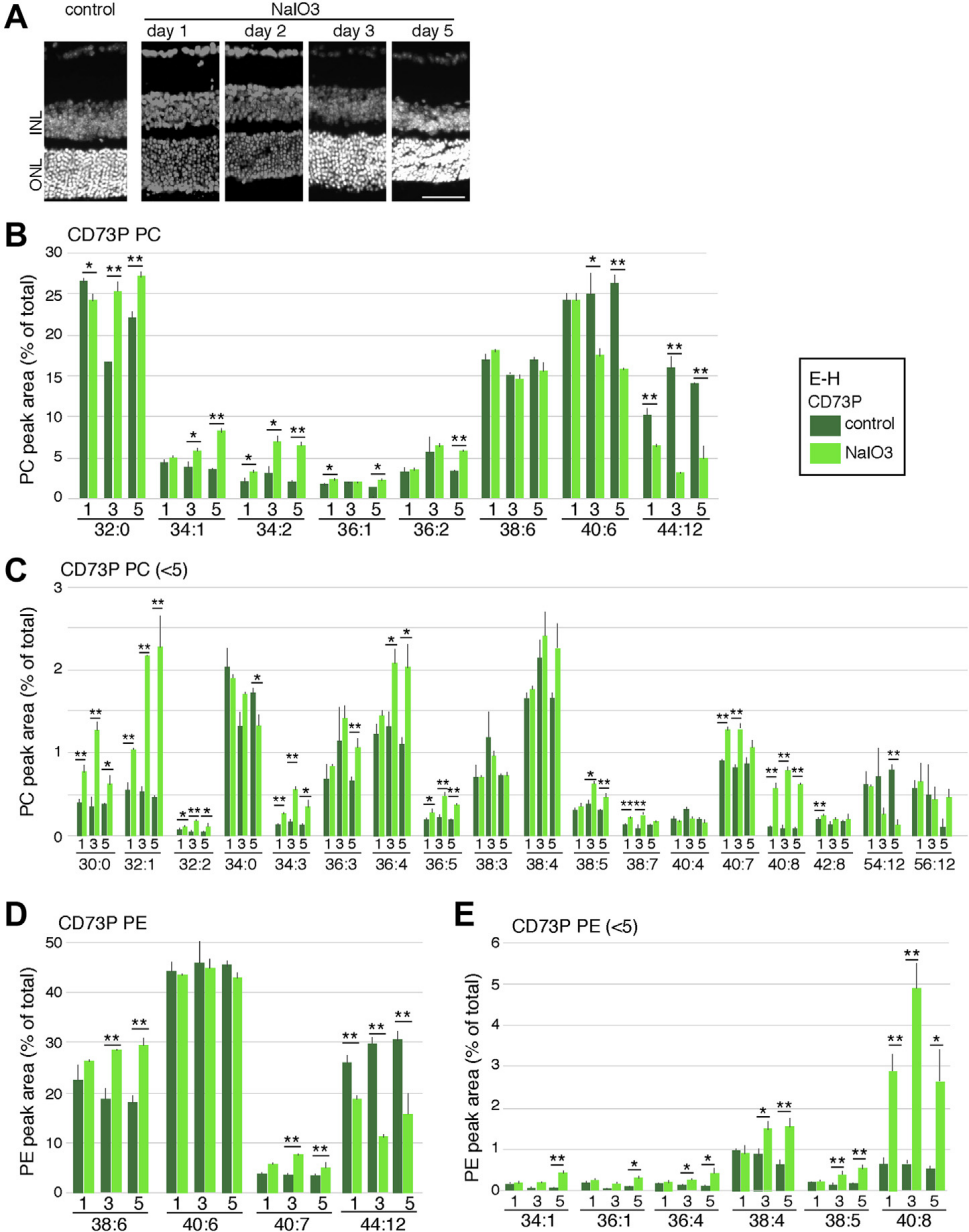
**PC and PE components of retinas after treatment with NaIO**_**3**_. *A* and *B*, adult mice were treated with NaIO_3_, and the retinas were harvested after 1, 3, and 5 days. The retinas were frozen sectioned and stained with 4′,6-diamidino-2-phenylindole to visualize the nuclei. The scale bar represents 50 μm. *B*–*E*, mice were treated with NaIO_3_, and the retinas were harvested at days1, 3, and 5. Harvested retinas were fractionated into CD73-positive and -negative populations, and then PC and PE components/5e4 cells were analyzed. The area of each species of PC (*B*, *C*) and PE (*D*, *E*) of CD73-positive fraction is shown. For PC, 43 species were detected, and the top 26 species are shown in (*E*) as the percent area of total 43 species area. For PE, 11 species were detected, and the top ten species are shown in *D* and *E* as the percent area of total 11 species area. The species that have averages of control/NaIO_3_ samples more than 5 in the CD73-positive sample are shown in (*B* and *D*). The species that have average of control/NaIO_3_ values less than 5 in CD73-positive sample are shown in (*C* and *E*). Values are average of three independent samples with standard deviation. ∗*p* < 0.05, ∗∗*p* < 0.01, by Student's *t* tests.

The difference of PC and PE levels of rd1 mice was partly explained by the difference of PC and PE species in photoreceptor and other cells, but we aimed to directly analyze the transition of PC and PE levels in the degenerating photoreceptor. For that purpose, we employed the sodium iodate (NaIO_3_) model because degeneration occurs more uniformly than in the rd1 model. NaIO_3_ causes photoreceptor degeneration, but it primarily targets the RPE (32). We examined PC and PE dynamism of CD73-positive and -negative cell fractions after treatment with NaIO_3_. We isolated mouse retinas 1, 3, or 5 days after treatment with NaIO_3_. Three days after treatment, the ONL of NaIO_3_-treated mice was distinguishable from that of control ONLs (Fig. 5*A*). Five days after treatment, ONLs in the NaIO_3_-treated mice were profoundly thinner than control ONLs (Fig. 5*A*).

Next, we examined the levels of PC and PE in CD73-positive and CD73-negative retinal cells in mice treated with NaIO_3_ (day 1, 3, and 5). When we analyzed the CD73-positive fraction, NaIO_3_ treatment decreased PC 44:12 significantly and increased PC 32:0 and 34:2. More than half of the PC species with small amount (Fig. 5*B*) showed increment, but it may be caused by the relative change caused by a large decrease of PC 44:12. In PE analysis, PE 44:12 decreased significantly, and the increment of PE 38:6 and 40:8 was remarkable (Fig. 5*D*). The relative levels of PC and PE species with small amount also showed changes of their percentage to the total amounts of PC and PE (Fig. 5, *C* and *E*).

The levels of most PC and PE species remained unchanged in CD73-negative cells after treatment with NaIO_3_, except for a slight decrease in PC 38:4 and 40:6 at day 3 (Fig. S4). Samples of day 1 and day 5 showed a similar pattern as that of day 3 sample (data not shown).

PC 34:1 and PE3 8:4 were most remarkably upregulated of their abundance in rd1 retina (Fig. 2, *E* and *F*), and the NaIO_3_-model data showed that both PC 34:1 and PE 38:4 were increased in the CD73-positive fraction.

To examine in more detail the pathological situation of NaIO_3_-induced retinal degeneration, we prepared samples for electron microscopic analysis. One day after NaIO_3_ administration, frozen section showed no obvious symptoms (data not shown) and electron microscopic analysis indicated a slight accumulation of fragmented OS in the border between OS and RPE (Fig. S5*A*). However, the gross morphology of OS itself was not different from that of the control (Fig. S6*B*). At day 2, the accumulated fragmented OS was increased, and the structure of RPE, such as microvilli, endoplasmic reticulum in cytoplasm, and basal showed apparent abnormality (Fig. S6*A*). OSs near the border of RPE showed a higher electron density, but those of other regions looked relatively normal with some disarranged region, which is assumed to be caused by invasion of the microglia (Fig. S6*B*). After 3 days, RPE cells started to disappear and large number of OS fragments had been accumulated in the subretinal space (Fig. S6*A*). At this stage, some OSs were apparently morphologically disordered and almost disappeared at day 5 (Fig. S6*B*). These results indicated that, at day 1 to day 3, the photoreceptors still retained a relatively normal morphology; however, the lipid composition had already started to change. In summary, NaIO_3_ treatment altered PC and PE levels in CD73-positive cells from the early phase of photoreceptor degeneration.

## Discussion

Although membrane lipid homeostasis regulates various biological processes in the retina, the lipid composition in different retinal cells and developmental stages is understudied. In this study, we used cell sorting and LC-MS to analyze the levels of PC and PE species in different cells in the developing retina under physiological and pathological conditions. Our analyses revealed dynamic changes in the lipid composition during development, and these changes were cell type specific. We also identified the acyl-chain of some PC species, and the acyl-chain composition was also developmental stage as well as cell type specific (Fig. S6).

The membrane content of both PC and PE dramatically increased during retinal development. Since the peak of rod photoreceptor birth is at around P0 to P2 (1), considering the complex membranous structure of photoreceptors, this observation may be explained by the maturation of rod photoreceptor during development, especially in the late stages of retinal development. However, the increase in rod photoreceptor numbers cannot explain the increase in PC and PE levels in the early stages of retinal development. Cone photoreceptors arise very early during retinal development; however, the increase in PC and PE levels early during retinal development may not be fully attributed to the increase in the numbers of cone photoreceptors since cone is a least population retinal subtype. Studies have shown that the composition of fatty acids changes drastically during brain development and the lipid profiles of growth cones and synaptosomes differ significantly (33). Considering that PC biosynthesis facilitates neurite outgrowth (34), neuron maturation during retinogenesis may affect the observed PC and PE levels. An in-depth examination revealed that the levels of various PC and PE species changed profoundly during retinal development in a cell type–specific manner. Although the majority of retinal cells are fully developed before P15, the levels of certain species, including PC 46:12, PC 54:12, PC 56:12, and PE 44:12, increased markedly between P15 and adulthood. The increase in these species may explain the steep increase in total VLC-PC and VLC-PE levels.

PC 34:1 and PE 38:4 were most remarkably upregulated of their abundance in rd1 whole retina (Fig. 2, *E* and *F*), and these two species are much more abundant in CD73-negative than CD73-positive cells, suggesting these changes may be caused by a relative decrease of rod photoreceptor in rd1 retina. However, NaIO_3_-treated mouse retina showed upregulation of these two species in CD73-positive cells, indicating that the change of these two species was partly explained by the increased CD73-negative cells but there must be specific increment of these two species in the rod photoreceptor after NaIO_3_ administration and ELOVL4 is essential for the synthesis of VLC-PC and VLC-PE in photoreceptors (13). Here, we showed that *Elovl2* and *Elovl4* were specifically expressed in photoreceptors. Furthermore, *Elovl2* was expressed in embryonic retina and *Elovl4* expression was increased after birth, suggesting that there is switching of the utilization of Elovl family members during differentiation. Furthermore, RNA-Seq data of Nrl-KO retina suggested that different species of Elovl family members are utilized between rod and cone. *Elovl2* and *Elovl5* are expressed in low level in normal retina, but cone-dominated retina showed strong expression of these two genes. In contrast, *Elovl4* expression was much lower in Nrl-KO retina. *Agpat3* and *Fads2* were also lower in cone-dominated retina than in normal retina, suggesting DHA metabolism is also different between cone and rod. In fact, higher levels of LC-PUFAs and VLC-PUFAs in rod-dominant retina than cone-dominant retina were reported (7), which was coincident with our prediction. Unexpectedly, *FADS2*, a desaturase also involved in EPA and DHA synthesis (13), was predominantly expressed in the embryonic retina, and its expression in mature rod photoreceptors was low (Fig. 4*A*). These findings suggest that DHA and EPA synthesis from α-linolenic acid (18:3) may be less significant and that the increased DHA levels in photoreceptors after birth may facilitate the rapid turnover of the OS in the adult retina (35). Studies in DHA-deficient rodents have indicated the requirement of dietary DHA to sustain the physiological DHA levels and retinal function (36).

In this study, we also showed that various PC and PE species differ significantly between rod photoreceptors and other retinal cells. Of note, the levels of PC 34:1, PC 36:1, PC 32:1, and PC 36:4 were significantly lower in rod photoreceptors than in other retinal cells, consistent with previous reports in the retinas of *Lpaat3* knockout mice (10). Since *Lpaat3* (*Agpat3*) mRNA levels were considerably higher in CD73-positive cells than in CD73-negative cells (Fig. 4*A*), we surmise that the differences in PC and PE species between CD73-positive and CD73-negative cells is primarily attributed to differences in the expression of critical enzymes involved in lipid metabolic pathways. A high level of *Lpaat3* transcripts was observed from P2, that is, early stage of rod differentiation, suggesting that the Lpaat3 plays pivotal roles in both differentiation and maintenance of rod cells.

Comparative analyses in membrane lipid composition in sorted cells and whole retinas revealed high similarities in E17 retinas. In adult retinas, despite the similar PC and PE levels between the two methods, the levels of long-chain fatty acids differed significantly. Since preparing single-cell samples from adult retinas is more difficult, and prolonged treatment with trypsin may cause cell death, specific retinal cell populations may have been lost. Despite this, retinal cell fractionation by cell sorting provided good-quality samples for subsequent LC-MS analyses, indicating that the combination of cell sorting and LC-MS is a promising approach to investigate the cell lineage–specific roles of lipid homeostasis.

Consistent with previous findings in *Lpaat3* knockout retinas (10), rd1 retinas exhibited a decrease in PC/PE 44:12 among other species during retinal development. This decrease in the levels of PC and PE species may be attributed to the gradual loss of photoreceptors in rd1 mice. NaIO_3_ treatment caused significant alterations in the levels of individual lipid species. Therefore, the relationship between RPE loss and lipid metabolism in photoreceptors merits further investigation.

## Experimental procedures

### Animals

All experiments adhere with the declaration of Helsinki and were approved by the Animal Care Committee of the Institute of Medical Science, University of Tokyo, and conducted in accordance with the ARVO (Association for Research in Vision and Ophthalmology) statement for the use of animals in ophthalmic and vision research. ICR mice and the rd1 mice were purchased from Japan SLC Co. The day when a vaginal plug was observed was considered as embryonic day 0 (E0), and the day of birth was termed as postnatal day 0 (P0). As “adult” mice, we used 8-week-old female ICR mice.

### NaIO_3_ administration to the mouse

As a photoreceptor degeneration model, administration of NaIO_3_ was employed. NaIO_3_ (Sigma-Aldrich) was administrated intravenously (tail vein) at 120 mg/kg, and the eyes were harvested after 5 days. PBS was administrated to control mice in both cases.

### Fluorescence activated cell sorting

Retinas were isolated from enucleated eyes and incubated with 0.25% Trypsin for 15 min to dissociate into single cells. Then, retinas were treated with 20% fetal bovine serum and DNaseI, dissociated into single cells, and stained with PE-conjugated anti-CD73 antibody (TY/23, BD) for 30 min on ice. Retinas were washed with 2% bovine serum albumin in PBS and stained with propidium iodide. CD73-negative and -positive cells were collected using FACSAria II (BD). For the experiments of Figure 3, unstained retinal cells were applied to FACSAria II and collected.

### Measurement of phospholipids from retina and retinal cells

Frozen retinas were sonicated in 70 or 100 μl of methanol. The suspensions were centrifuged at 10,000*g* for 5 min and the collected supernatant was diluted with methanol for phospholipid analysis. Sorted cells from retinas were suspended in 50 μl of methanol. The cell suspensions were centrifuged at 10,000*g* for 5 min and the collected supernatant was used for phospholipid analyses. LC-selected reaction monitoring (SRM)-MS analysis was performed using a Nexera UHPLC system and triple quadrupole mass spectrometers LCMS-8050 (Shimadzu Corporation). For separation, an Acquity UPLC BEH C8 column (1.7 μm, 2.1 mm × 100 mm, Waters) was used with the following ternary mobile phase compositions: 5 mM NH_4_HCO_3_/water (mobile phase A), acetonitrile (mobile phase B), and isopropanol (mobile phase C). Either one of following programs of the pump gradient [time (%A/%B/%C)] was used: 0 min (50/45/5)-10 min (20/75/5)-20 min (20/50/30)-27.5 min (5/5/90)-28.5 min (5/5/90)-28.6 min (50/45/5), or 0 min (75/20/5)-20 min (20/75/5)-40 min (20/5/75)-45 min (5/5/90)-50 min (5/5/90)-55 min (75/20/5). The flow rate was 0.35 ml/min, and column temperature was 47 °C. The injection volume was 5 μl. SRM analysis with phospholipid class discrimination was performed with the following transitions: [M + H]^+^→184 for PC, [M + H]^+^→[M+H-141]^+^ for PE. Peak areas of all identified species were summed to obtain the total signal. Peak areas of individual species were normalized with this sum and are illustrated as precent total.

Molecular species show that PC (x:x) are the number of carbon chain and double bond after PC shows sum of two acyl chains.

### Characterization of the acyl-chain composition of DHA-containing PC species

To characterize the acyl-chain compositions of DHA-containing PC species, targeted LC-SRM-MS/MS using negative ion mode was performed on PC 38:6, 40:6, 44:12, 46:12, 56:12. The fragment ion signals of possible fatty acyl chains were obtained by the following transitions: [M + HCO_3_]^-^ → [FA-H]^-^, where [FA] is the monoisotopic mass of each fatty acid. The following acyl chains were targeted: C16:0 (*m/z* 255.25), C16:1(*m/z* 253.25), C18:0(*m/z* 283.25), C18:1(*m/z* 281.25), C18:2(*m/z* 279.25), C20:2(*m/z* 307.25), C20:3(*m/z* 305.25), C20:4(*m/z* 303.25), C20:5(*m/z* 301.25), C22:5(*m/z* 329.25), C22:6(*m/z* 327.25), C24:5(*m/z* 357.25), C24:6(*m/z* 355.25), C34:6(*m/z* 495.25). Of note, *m/z* 283.25 may contain signals of C22:6 in addition to C18:0; however, this did not hinder acyl chain identifications of target PC species.

### Protein concentration determination

The retinas and cell pellets after lipid extraction were evaporated and resuspended with a solubilizer containing 8 M Urea, 2 M thiourea, and 2% CHAPS and 0.8% Triton X-100. The samples were solubilized using a sonicator before protein concentration determination. The protein concentrations were determined by the bicinchoninic acid protocol by using Pierce 660nm Protein Assay (Thermo Fischer).

### Immunohistochemistry

Immunohistochemistry of frozen-sectioned retina was performed as described (37). Nuclei were visualized with 4′,6-diamidino-2-phenylindole (1 μg/μl) staining. Primary antibodies were: mouse IgG anti-GFAP (1:500, Sigma-Aldrich) and rabbit anti-Iba1 (1:2000, FUJIFILM Wako Pure Chemical) antibodies, and primary signals were visualized with appropriate secondary antibodies conjugated to Alexa Flour 488 or Alexa Flour 594 (Molecular probes).

### Transmission electron microscopy

Eyes were enucleated, immediately cut into hemispheres in a solution containing 2% paraformaldehyde and 2.5% glutaraldehyde, and incubated in the same fixative for 2 h. The samples were then postfixed in a solution containing 2% OsO4, dehydrated in a graded series of ethanol, and embedded in Epon 812 resin mixture (TAAB Laboratories Equipment Ltd). Ultrathin sections were created using an ultramicrotome, stained with uranyl acetate and lead citrate, and examined under an electron microscope (JEOL JEM-1400Flash).

### Statistics

*p* Values [∗*p* < 0.05, and *p* > 0.05 (not significant)] were calculated by the Student's *t* test or Tukey HSD (honestly significant difference) test.

## Conflict of interest

The authors declare that they have no conflicts of interest with the contents of this article.
