# Supplementary material for: Mapping membrane lipids in the developing and adult mouse retina under physiological and pathological conditions using mass spectrometry
Source: J Biol Chem. 2021 Jan 16;296:100303. doi: 10.1016/j.jbc.2021.100303 (PMC7949107; doi:10.1016/j.jbc.2021.100303)

Mapping of membrane lipids in the developing and adult mouse retina under physiological and pathological conditions using mass spectrometry

Authors: Fumie Hamano, Hiroshi Kuribayashi, Toshiro Iwagawa, Katsuyuki Nagata, Takao Shimizu, Hideo Shindou, and Sumiko Watanabe

Supporting Fig. 1

Transition of PC and PE components during retinal development

Mouse retinas at different developmental stages were isolated, and PC and PE composition were analyzed by LC-MS. VLC-PC with average values less than 4e7 populations are shown. Values are area/ng protein, and average of 3 independent samples with standard variation.

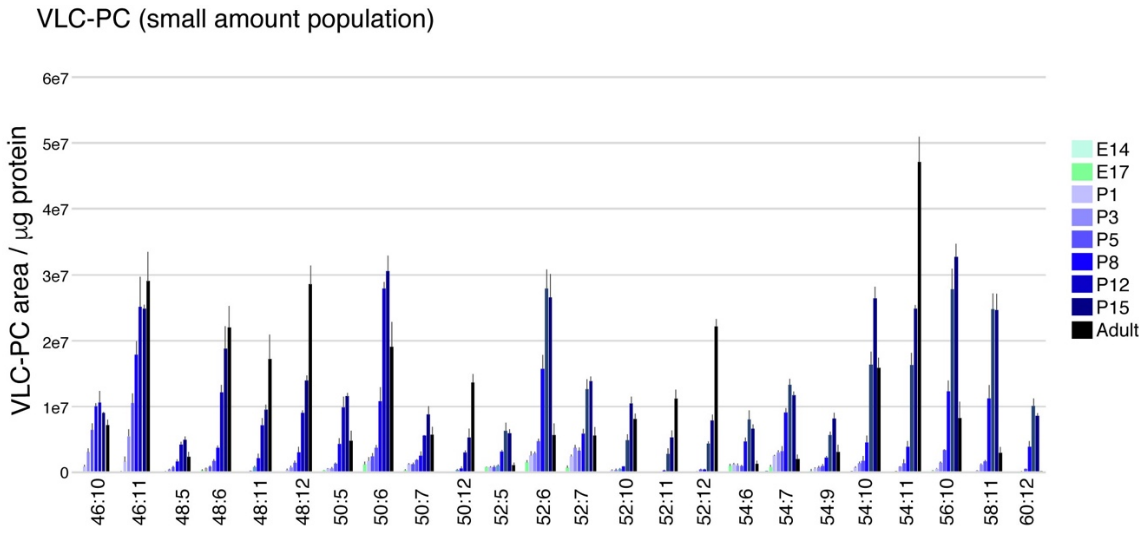

Supporting Fig. 2

PC and PE components of the cell sorted retinal cells are shown. Mouse retinal cells isolated from E17 embryo or adult were treated with trypsin, then the cells were applied to a cell sorter, and different number of cells from 5e3 to 1e5 were collected (A). Then cells were applied to LC-MS to analyze PC (B, C, F, G) and PE (D, E, H, I). At the same time, lipids fractions extracted from whole retinas, which were not treated with trypsin from E17 (F, H) or adult (G, I) mice were analyzed of PC and PE as controls (A). B and D show total area of PC (B) and PE (D). C and E show total area corresponding equivalent to 1e4 cells of each sample of PC (C) and PE (E). F-I show area of each species of PC of PE to the total area (%) of E17 (F, H) and adult (G, I). The PC species which have more than 1 (%) value of average are shown in left panels of F and G. Those with from 0.5 to 1 are shown in right panels of F and G. Values are average of 3 independent samples with standard deviation for the sorted cell samples. For E17 and adult whole retina control samples, values are representative one sample.

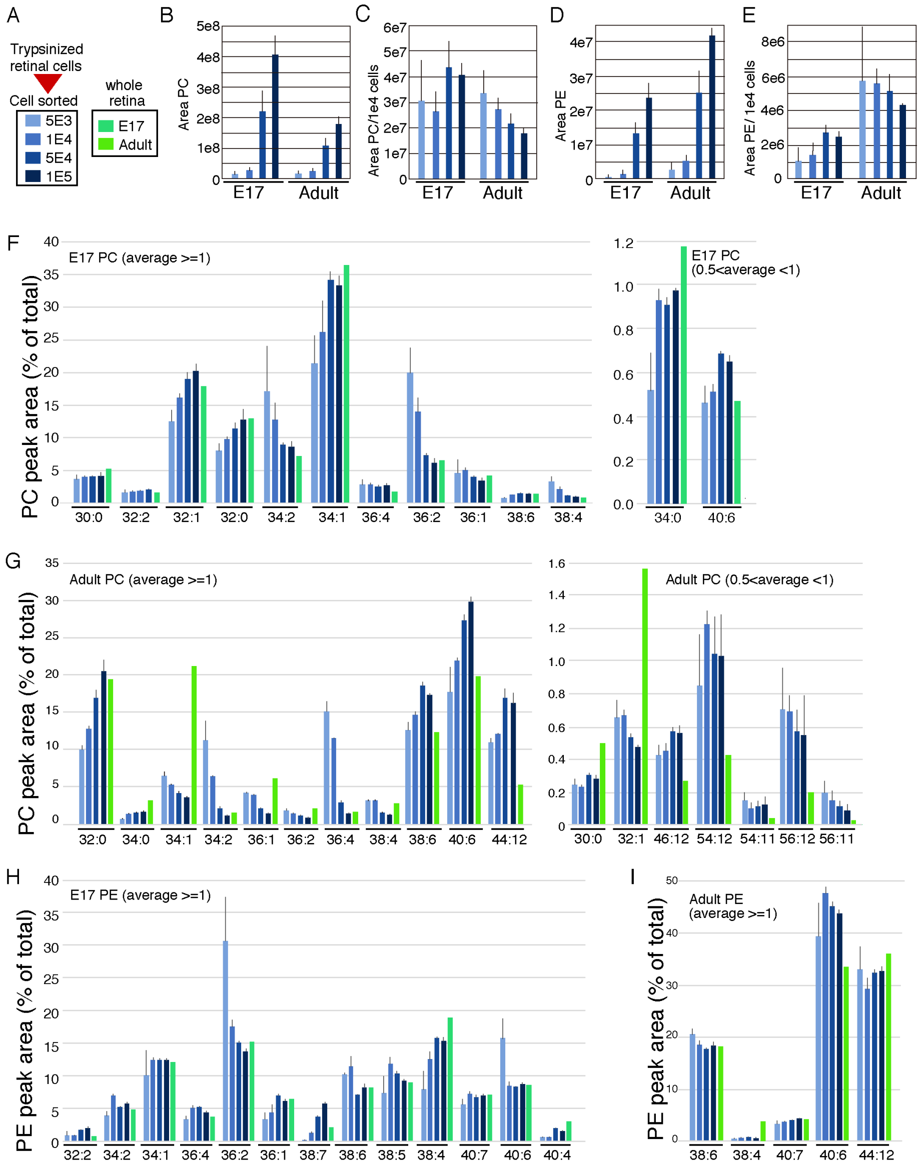

Supporting Fig. 3

Expression of transcripts of retinal subtype specific genes; *Math3* (amacrine), *Vsx2* (bipolar), *Timp1* (Müller glia), and *Nrl* (rod photoreceptor) in developing retina. Values are FPKM of RNA-seq data (GSE71462, GSE71464).

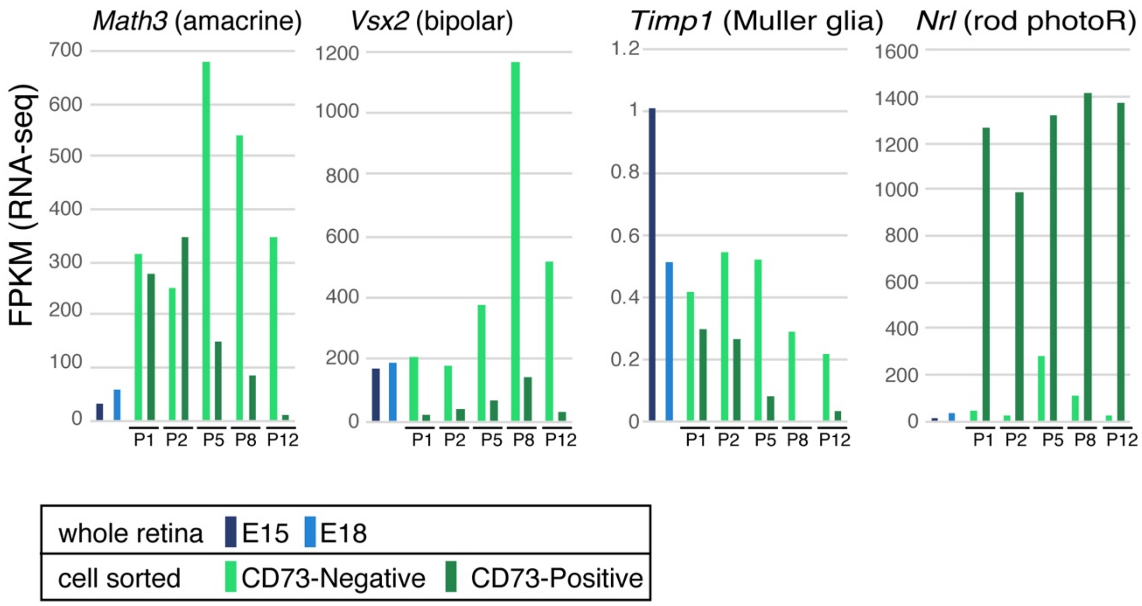

Supporting Fig. 4

PC and PE components of CD73 negative fraction of retinas after treatment of MNU or NaIO3 are shown. Adult mice were treated with MNU or NaIO3, and the retinas were harvested after 3 or 5 days, respectively. The retinas were fractionated into CD73-potive and -negative populations, and PC and PE components were analyzed. Area of each species of PC (A) and PE (B) of CD73 negative fraction are shown. The species which has average of control, MNU and NaIO3 values more than 5 are in the left panes, and that with less than 5 are in the right panels. Values are average of 3 independent samples with standard deviation. * p<0.05, ** p<0.01, by Tukey HSD test.

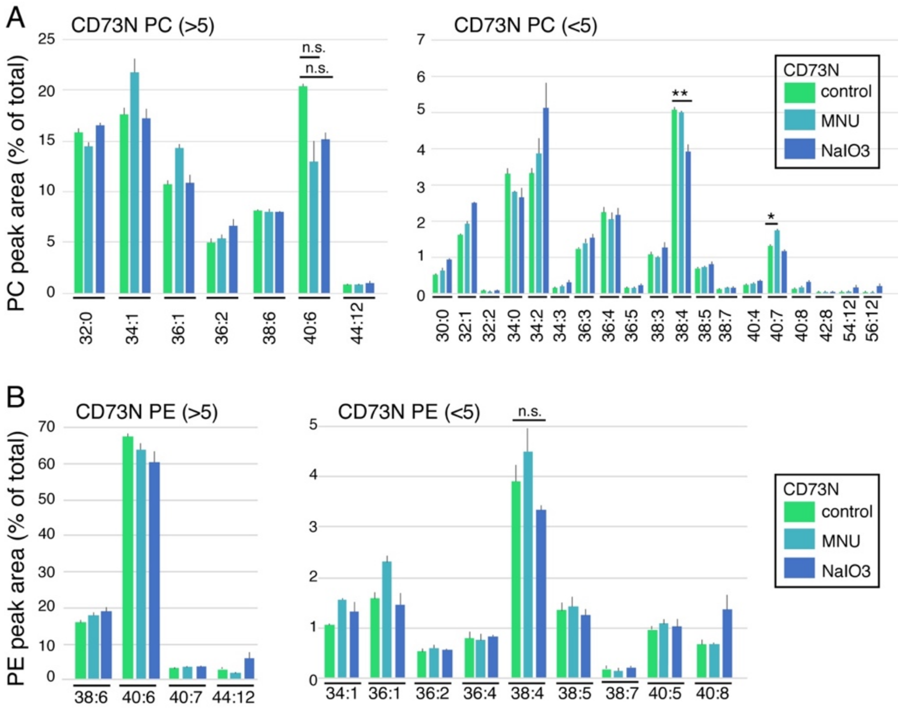

Supporting Fig. 5

Electron microscopic analysis of retinas from control and NaIO3 administrated mice. RPE and outer segment regions are shown. Electron dense fragmented outer segments (red arrow) are accumulated between intact OS and RPE. Scale bar: 5 μm

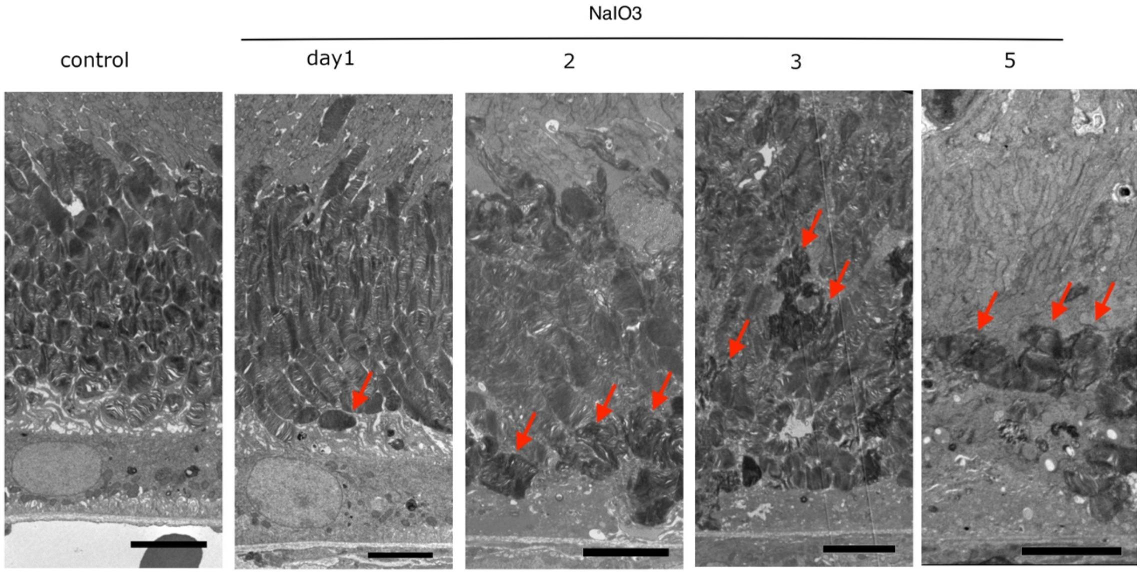

Supporting Fig. 6

Identification of the acyl-chain of PC38:6, PC40:6, PC44:12, PC46:12, PC54:12, and PC56:12 were performed by targeted LC-SRM-MS/MS using negative ion mode. Representative data; PC38:6 (A) and PC40:6 (B) are shown. Left panels show MS-chromatograph of the acyl-chain 16:0, 18:2, 20:4, and 22:6 (A), and 16:0, 18:0, 20:2, 20:4, 22:6, and 24:6 (B). The right graphs show strength of signals of acyl-chain fragments. DHA fragment is indicated in magenta (A) and in brown (B).

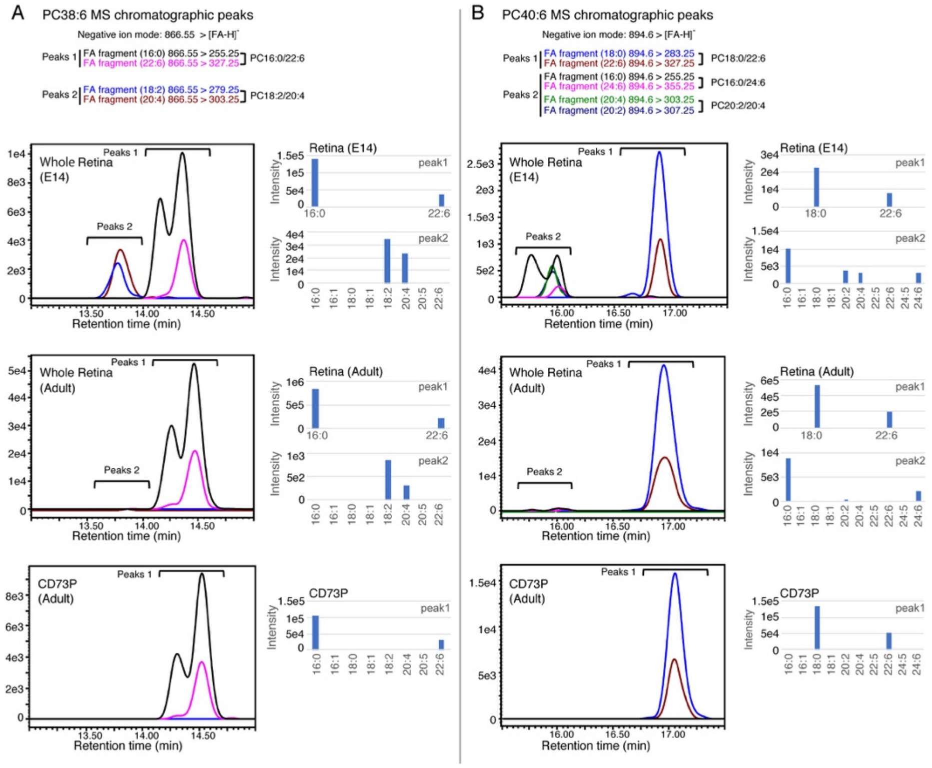

Supplement: Supplementary file 1 — Figures S1 to S6 [file mmc1.pdf]
